# Forensic Aftercare Facilities and Their Impact on the Releasability of Persons Who Committed Sexual Offenses: A Three Group Comparison

**DOI:** 10.1177/0306624X241246519

**Published:** 2024-04-27

**Authors:** Julia Sauter, Agne Mauzaite, Tatjana Voß, Joanna Vogel

**Affiliations:** 1 Institute of Psychology, University of Kassel, Kassel, Germany; 2 Institute of Psychology, Fernuniversität Hagen, Hagen, Germany; 3 Charité—Universitätsmedizin Berlin, Corporate Member of Freie Universität Berlin, Humboldt-Universität zu Berlin, and Institute of Health, Institute of Forensic Psychiatry, Berlin, Germany; 4 Department of Health Science, IB University of Applied Social Sciences, Berlin, Germany

**Keywords:** sex offender, offender treatment, forensic follow-up treatment, aftercare, release options, sexual offenses

## Abstract

Partly due to a lack of release options for individuals who committed sexual offenses, forensic follow-up treatment has been strengthened latest since 2007. The current study investigates whether the foundation of a professionalized follow-up-treatment has actually improved release options for individuals who committed sexual offenses. Thus, the aim of the present study was to assess the difference in criminogenic needs and recidivism relevant characteristics (e.g., index offense, criminal history, psychiatric diagnoses and risk assessment) between three groups who had been released from forensic psychiatry at different times or under different outpatient follow-up modalities: (1) individuals released prior the foundation of professionalized follow-up-treatment, (2) individuals released after the foundation and received treatment, and (3) individuals released after the foundation but not receiving this special treatment. It was found that with the availability of professionalized forensic followup treatment, persons with higher scores in common risk assessment tools and a longer duration of implacement had been released. Indeed, this indicates an increased risk tolerance among decision makers. However, it was not those who were released after the foundation of the professionalized forensic follow-up treatment but without this specific treatment who showed the lowest initial risk, but those who were released prior to the foundation. Results are discussed in terms of possible explanations and methodological issues.

## Introduction

A release from closed forensic institutions of individuals who committed sexual offenses can be considered as a weight decision between manageable and acceptable risk to society, on the one hand, and the right to rehabilitation and reintegration into society for the convicted individual, on the other. The assessment as to what constitutes a still acceptable risk of recidivism is a subjective one (Rettenberger et al., 2017), and is known to be undergoing change, partly determined by the criminal policy and social climate. In particular committing of a sexual offense has for some time been considered as a predictor of unfavorable release prospects (Schalast et al., 2007). Stigma for individuals with pedophilic interest is tremendous and even higher for individuals convicted of a sexual offense. In two surveys 14% respectively 27% of participants agreed that individuals with a pedophilic disorder should better be dead, even if they never had committed criminal acts, and only 10% respectively 6%would except these persons in their neighborhood (Jahnke et al., 2015). So overall, there seems to be limited risk tolerance in society when it comes to release options for individuals who committed sexual offenses. Simultaneously, and in line with these findings, some releases failed because it was not possible to create a sound release environment for those to be released for the time after imprisonment or HTO (Marquant et al., 2018; Schalast et al., 2007). As a result, but also due to the overcrowding of inpatient forensic facilities (Leygraf, 2006), more and more forensic outpatient clinics were established from the mid-2000s onward. In 2007, professional forensic therapeutic aftercare was legally established in Germany (Bundesgesetzblatt, 2007, I, p. 513), which led to a progressive expansion of forensic aftercare. Purpose of forensic outpatient follow-up treatment is to provide a setting in which skills learned in inpatient setting can be proved, tested, stabilized, and further developed (Freese, 2003; Zisterer-Schick, 2011). Through the dual function of support and control, they are intended to reduce the likelihood of recidivism, particularly for those released who have committed a sexual offense.

Overall, individuals who have committed sexual offenses are not very likely to recidivate with a sexual offense. Hanson and Bussière (1998) found in their meta-analysis a rate of 13.4% after a follow-up period of 4 to 5 years. This result has been replicated many times over the years. In 2009, Hanson et al. (2009) reported a recidivism rate of 10.9% for treated and 19.2% for untreated individuals who offended sexually. And the recent meta-analysis by Schmucker and Lösel (2017) also reported a recidivism rate of 10.1% in treated and 13.7% in untreated individuals. It should also be noted, however, that the likelihood of recidivism increases in the presence of sexual deviancy and antisocial orientation, and that the recidivism rate regarding to nonsexual offenses was also much higher in this group (36.2%, Hanson & Morton-Bourgon, 2005; 31.8% vs. 48.3%, Hanson et al., 2009). In contrast to violent offenders, this risk of recidivism appears to persist after release and in the subsequent years (Hall, 1995).

Due to methodological difficulties, the effectiveness in terms of reducing recidivism of forensic (outpatient) treatment cannot be confirmed with sufficient certainty. For legal as well as ethical reasons it is mostly not possible to assign treatment randomly. Accordingly, Schmucker and Lösels (2017) meta-analysis of the effectiveness of treatment for individuals who committed sexual offenses, considering methodological quality standards, is yet based on 29 studies, of which only 6 had a randomized sample design. Most included studies were conducted in North America (19 of 29). Overall, there was a significant reduction in recidivism rates associated with treatment (the odds ratio was 1.41 lower for treated individuals compared to untreated individuals for recidivating with a sexual offense). The effectiveness of outpatient treatment was examined in 12 of the 29 studies. Overall, they appeared to be more effective than inpatient treatment (Schmucker & Lösel, 2017; first meta-analysis from Lösel & Schmucker, 2005). A similar picture emerges for the studies conducted in Germany. With overall mixed methodological quality, nine studies of outpatient forensic treatment were identified whose overall lower recidivism rates give cause for cautious optimism (Sauter et al., 2017).

All studies included in reviews and meta-analyses so far have focused on the comparison of recidivism rates between released offenders who received (further outpatient) treatment and those who received no or another form of forensic follow-up care. However, the question of whether the establishment of forensic follow-up treatment in itself has improved the release options for individuals who committed sexual offenders has hardly been investigated. Thus, the aim of the present study was to assess the difference in criminogenic needs and recidivism relevant characteristics between persons who have committed a sexual offense and who could be released prior to the foundation of professionalized forensic follow-up care and those who could be released since and with professionalized forensic follow-up treatment then available. In addition, those receiving outpatient follow-up treatments were compared with those who were released from forensic hospitals after foundation but without professionalized forensic follow-up. If the establishment of forensic follow-up care contributed to an improvement of release options, those released via professionalized forensic follow-up treatment should have the highest scores associated with recidivism, whereas the other two groups should differ only marginally.

## The Forensic Therapeutic Outpatient Clinic in Berlin

While the academic debate on the effectiveness of outpatient forensic aftercare continues, in practice, as demanded by policy makers and society, forensic aftercare facilities are being established and conceptually expanded. Different countries have found different strategies to pursue the public safety goal when releasing offenders from closed forensic facilities. These differences are mainly due to different legal frameworks as well as historical backgrounds (Gordon & Lindqvist, 2007). In Germany, individuals who have committed a sexual offense and who have been found not or partly not guilty by reason of insanity (§20, 21 of German penal code) can be administered to a forensic hospital and treated under a hospital treatment order (HTO). An HTO is potentially indefinite until its release is confirmed by a court. In most cases, this is done with the involvement of a forensic expert opinion concerning the question of criminal prognosis. Therefore, special forensic aftercare outpatient clinics were established to improve the criminal prognosis through an improved release setting (Freese, 2003; Müller-Isberner, 1996; Warmuth, 1990).

In Berlin, Germany’s Capital, the Forensic Therapeutic Outpatient Clinic for released persons who committed serious violent and/or sexual offenses (dt.: Forensisch-Therapeutische Ambulanz für Gewalt- und Sexualstraftäter, FTA) was established in 2005, involving psychiatric, psycho-therapeutical and social work services, with most patients seen on a 14-day basis. Since treatment with testosterone-lowering medication was increasingly administered under a HTO in Berlin starting around 2007 (Sauter et al., 2021), a professional follow-up was needed that would be able to continue this off-label treatment on an outpatient basis after release. In 2010, the FTA received authorization for this very purpose (Sauter et al., accepted). In terms of effectiveness of the FTA, a quasi-experimental study conducted in 2015 provides optimism. Using a comparison group matched by criminogenic markers, which had been released without treatment by the FTA, it was shown that although persons who had received treatment by the FTA recidivated significantly less during the follow-up treatment period, they adjusted to a similarly high recidivism rate after treatment (Sauter et al., 2015).

## Aim of the Present Study

Aim of the present study was to investigate whether the foundation of the FTA was associated with an improvement in release prospects for persons who committed sexual offenses. The group of sexual offenders is particularly suitable as they are considered the most difficult group of offenders to release due to their high level of stigmatization (Jahnke et al., 2015; Schalast et al., 2007). Thus, three study groups were compared in the present study: (1) those who were released prior the foundation of the FTA, (2) those who were released after the foundation of the FTA and were provided follow-up treatment by the FTA, and (3) those who were released after the foundation of the FTA and were not provided follow-up treatment by the FTA. Our hypothesis was that those released via the FTA would have the more serious offenses, more criminal convictions, longer terms of detention, the highest Static-99R score, and the highest psychological salience, compared to those released prior to the existing option of professionalized forensic follow-up treatment. With this in mind, we assumed that those persons who had been released since the foundation of the FTA, have not received FTA support, were the group with the lowest initial risk of recidivism.

## Methods

### Study Design and Procedure

The study represents a classic comparison group design. Using a retrospective file analysis, data were collected of all persons who had committed a sexual offense and have been released from a HTO between January 1st, 1997 and June 30th, 2016 in Berlin. The persons were then divided into the three study groups: (1) Persons who were released from HTO prior to the foundation of the FTA on July 1st, 2005, that is, at a time when forensic follow-up treatment by this professionalized outpatient clinic could not yet be implemented (comparison group pre FTA [CG_pre]: released between January 1st, 1997 and July 1st, 2005); (2) persons who received forensic outpatient follow-up treatment by the FTA after their release from HTO (treatment group FTA [TG_FTA]: released between July 1st, 2005 and June 30th, 2016); and (3) persons who were released from HTO after the foundation of the FTA but who did not receive follow-up treatment by the FTA (comparison group post FTA [CG_post]: released between July 1st, 2005 and June 30th, 2016). Group statistical methods were used to compare the three groups. On the one hand, it was to be investigated whether the treatment group TG_FTA) in contrast to the comparison group (CG_pre) was in fact the more unlikely group to be released from HTO. On the other hand, it should also be ensured that the group of persons considered to be at higher risk has actually been released via the FTA. Therefore, both groups, the CG_pre and the CG_post, were examined in relation to the treatment group TG_FTA.

The ethics committee at the Charité – University Medicine, Berlin, approved the present study (EA 1/210/18).

### Measures

#### Retrospective File Analysis

Data on criminal history and index offenses were collected from court decisions and Federal Central Registers. Since persons had already been released, there was no personal contact during the data collection. Psychiatric diagnoses according to ICD-10 were therefore taken from the most recent medical record or expert opinions for court cases.

#### Static-99R

(Phenix et al., 2016) is the revised version of the Static-99 (Hanson & Thornton, 2000), the most commonly used risk assessment instrument for adult males who have committed a sexual offense. Static-99R was chosen to objectively measure the initial risk of recidivism. It entails 10 mostly static items (e.g., Age, partnerships over 2 years, type and number of prior convictions, victim variables, etc.). Scores can range from −3 to 12 points and are transferred into nominal risk levels from “very low risk” (equivalent to −3 and −2 points) to “well above average risk” (equivalent to 6 points and larger). Compared to the Static-99, the Static-99R weights age at the time of the prognosis more heavily (Helmus et al., 2012). The Static-99R is considered a reliable and valid risk assessment instrument for recidivism with a sexual or violent offense after a 5-year follow-up period with predictive validities ranging from Area under the Curve (AUC) = 0.720 to AUC = 0.715 (Helmus et al., 2012). Interrater reliability is known to be high (e.g., intraclass correlation coefficient [ICC] = 0.89 (McGrath et al., 2012). For the present study, the Static-99R was rated for all persons by one of the authors, who was intensively trained.

### Participants

**Figure 1.**
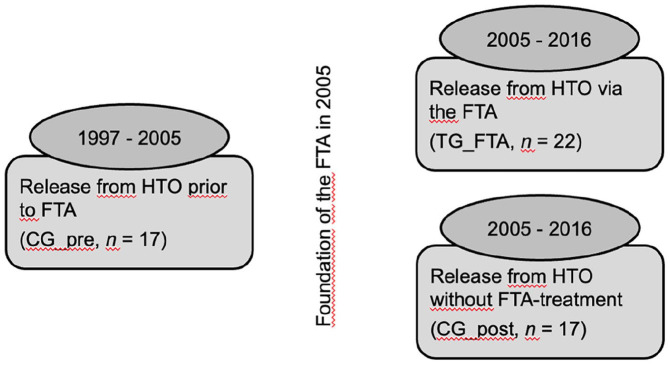
Study groups: (1) Comparison group released prior to the foundation of the professionalized forensic follow-up treatment between 1997 and 2005 (i.e., CG_pre, *n* = 17); (2) Treatment group released via the FTA between 2005 and 2016 (i.e., TG_FTA, *n* = 22); (3) Comparison group released after the foundation of the FTA but without FTA-Treatment between 2005 and 2016 (i.e., CG_post, *n* = 17).

From a total of *N* = 2.260 files, *n* = 56 (2.5%) persons who committed a sexual offense and were released from HTO between January 1st, 1997 and June 30th, 2016 could be identified. Before the foundation of the FTA on July 1st, 2005, *n* = 17 persons could be released from HTO (CG_pre), since its foundation *n* = 22 could be released via the FTA (TG_FTA) and another *n* = 17 have been released after the foundation of the FTA but without FTA-Treatment (CG_post, see Figure 1).

### Statistical Analyses

To calculate differences between the study groups *χ*^2^-analyses and *t*-tests for independent samples were used. Mean differences between all three study groups were calculated using one-way ANOVAs. In the case of violations of the assumption of homogeneity of variance, corrected Welch’s *F* was used (Welch, 1951). Since it is less sensitive to bias, omega squared *ω*^2^ was calculated as effect size (Hays, 1963). According to Kirk (1996), values of 0.01 were interpreted as small, of 0.06 as medium and of 0.14 as large effects. Games-Howell was used as a post hoc test to compare the means of the three study groups, as not all population variances were equal (Field, 2018). For all tests, alpha level was set at *p* < .05. Statistical analyses were performed using SPSS Statistics for Windows, Version 24.0 (Armonk, NY: IBM, 2016).

## Results

### Index Offense and Criminal History

#### Index Offense

**Figure 2.**
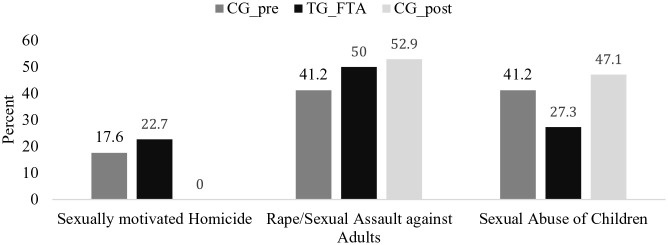
Kind of index offense of the comparison group prior to the foundation of the FTA (CG_pre; *n* = 17), Treatment Group FTA (TG_FTA; *n* = 22) and the Comparison Group released post FTA but without FTA-Treatment (CG_post; *n* = 17).

There was no significant difference regarding the kind of index offenses committed between those released from HTO prior to the foundation of the FTA (CG_pre) and the treatment group TG_FTA (see Figure 2; *Fisher’s Exact Test* = 0.888, *Cramer’s V* = 0.15, *p* = .697). Of the persons released prior to the foundation of the FTA (CG_pre), *n* = 3 (17.6%) had committed a sexually motivated homicide, *n* = 7 (41.2%) rape or sexual assault against adults and *n* = 7 (41.2%) sexual abuse of children. Of those released via the FTA (TG_FTA), *n* = 5 (22.7%) had committed a sexually motivated homicide, *n* = 11 (50%) rape or sexual assault against adults, and *n* = 6 (27.3%) sexual abuse of children.

After the foundation of the FTA, none of those who had committed a sexually motivated homicide were released without FTA-Treatment (TG_FTA: *n* = 5 vs. CG_post: *n* = 0). On the opposite, more of those who had committed sexual abuse of children were released from the HTO without further treatment by the FTA (TG_FTA: *n* = 6 vs. CG_post: *n* = 8). Nevertheless, no significant difference was found in this respect either (*Fisher’s Exact Test* = 4.783, *Cramer’s V* = .36, *p* = .089). With regard to rape or sexual assault against adults, there was hardly any difference between the two study groups (TG_FTA: *n* = 11; CG_post: *n* = 9).

However, there were differences in the prison sentence ordered by court, which can be considered as a measure of the severity of the offenses committed (see Table 1). With an average prison sentence of *M* = 7.3 years, the TG_FTA showed an above mean difference of *M*_Diff_ = 2.9 years, *p* = .211 compared to the CG_pre, and an *M*_Diff_ = 4.0, *p* = .037 compared to the CG_post. At the time of the index offense, persons of the TG_FTA were the youngest, yet, this difference was not significant, TG_FTA – CG_pre: *M*_Diff_ = −2.6, *p* = .627, TG_FTA – CG_post: *M*_Diff_ = −7.1, *p* = .306.

**Table 1.** Differences Between the Three Study Groups Regarding Their Criminal History and Standardized Recidivism Risk using Static-99R (CG_pre: *n* = 17, EG_FTA: *n* = 22, CG_post: *n* = 17).

	CG_pre		TG_FTA		CG_post		Comparison		
	*M* (*SD*)	95%-CI	*M* (*SD*)	95%-CI	*M* (*SD*)	95%-CI	*F* ^a^	*df_M_/df_R_*	*ω* ^b^
Age at index offense	32.0 (8.3)	[27.7, 36.3]	29.4 (9.1)	[25.4, 33.4]	36.5 (17.7)	[27.4, 45.7]	1.246	2/32	.02
Prison sentence^2^	4.4 (2.8)	[2.4, 6.4]	7.3 (6.0)	[4.3, 10.3]	3.2 (1.4)	[1.9, 4.5]	3.634	2/20	.07*
Age at first offense	24.8 (9.1)	[20.1, 29.5]	20.0 (6.7)	[17.0, 23.0]	31.8 (18.8)	[22.1, 41.4]	3.997	2/29	.11*
Previous convictions	3.5 (3.8)	[1.5, 5.4]	4.6 (3.3)	[3.2, 6.1]	1.8 (2.3)	[0.7, 3.0]	3.659	2/53	.09*
Sexual offenses	0.8 (1.4)	[0.1, 1.6]	1.6 (1.7)	[0.8, 2.3]	0.7 (1.2)	[0.1, 1.3]	1.864	2/53	.03
Violent offenses	0.7 (2.0)	[0, 1.7]	0.3 (0.6)	[0.0, 0.5]	0.3 (0.5)	[0.1, 0.5]	0.383	2/30	.01
Total length of PPS	2.6 (3.7)	[0.7, 4.5]	4.6 (5.1)	[2.3, 6.9]	0.7 (1.1)	[0.2, 1.3]	7.224	2/28	.12**
HTO treatment duration	8.5 (5.7)	[5.5, 11.4]	16.1 (6.2)	[13.4, 18.9]	12.9 (9.6)	[7.9, 17.8]	5.352	2/53	.13**
Age at release	41.0 (9.9)	[35.9, 46.1]	45.8 (10.7)	[41.0, 50.5]	50.2 (19.4)	[40.3, 60.2]	1.899	2/32	.03
Static-99R (Release)	3.9 (1.7)	[3.0, 4.8]	5.6 (2.1)	[4.7, 6.6]	4.3 (1.9)	[3.3, 5.3]	4.514	2/53	.11*

*Note. CG_pre* = Comparison Group via the foundation of the FTA; *TG_FTA* = Treatment Group with Treatment in the FTA; *CG_post* = Comparison Group post foundation of the FTA but without FTA-treatment; *Prison Sentence*: Actual Prison Sentence in years; *Total Length of PPS* = Total Duration of all previous Prison Sentences in years; *HTO Treatment Duration* = Duration of the actual Treatment under a Hospital Treatment Order in years. *M* = mean; *SD* = standard deviation; 95%-CI = 95%-confidence interval; *df_M_* = degrees of freedom for the model/effect; *df_p_* = degrees of freedom for the residual sum of squares; ***ω***^2^: effect size omega squared.

a In the case of violations of the assumption of homogeneity, *Welch’s F* was used.

b Since a prison sentence had not been ordered against all individuals, the sample size was reduced here: CG_pre: *n* = 10, TG_FTA: *n* = 18, CG_post: *n* = 7.

* p < .05. **p < .01. ***p < .001.10 (two-tailed).

#### Criminal History

With a view to criminal history, there were some significant differences between the three study groups (see Table 1). At the time of their first offense, persons of the TG_FTA were the youngest, followed by persons of the CG_pre, and the CG_post. A closer look at the three study groups shows that TG_FTA did not differ significantly from CG_pre (*M*_Diff_ = −4.8, *p* = .176), but tends to differ from CG_post (*M*_Diff_ = −11.8, *p* = .058). In the same order, the persons of the TG_FTA showed the largest number of previous convictions. While the difference to CG_pre was not significant (*M*_Diff_ = 1.2, *p* = .584), there was a significant difference to CG_post (*M*_Diff_ = 2.8, *p* = .009). No significant differences were found regarding the type of criminal record, that is, sexual offenses and violent offenses committed before the index offense.

As a measure of the severity of the offenses committed so far, the total duration of all prison sentences ordered against the persons was calculated. In line with the results reported earlier, it was found that the total length of all previous sentences was highest in the study group TG_FTA. This medium effect (*ω*^2^ = .12) can mainly be attributed to the difference between TG_FTA and CG_post (*M*_Diff_ = 3.9, *p* = .006), whereas TG_FTA and CG_pre did not differ significantly from each other (*M*_Diff_ = 2.0, *p* = .330).

### Treatment Under the Last HTO and Standardized Risk Assessment using Static-99R

On average, none of the study groups was as long under detention as the TG_FTA (see Table 1). In total, the TG_FTA was treated *M*_Diff_ = 7.7 (*p* = .001) years longer under HTO than the CG_pre, and *M*_Diff_ = 3.2 years (*p* = .457) longer than the CG_post. At the time of release from the HTO, the TG_FTA revealed the highest Static-99R score with an average score of *M* = 5.6. This score was *M*_Diff_ = 1.8 points above CG_pre (*p* = .018) and *M*_Diff_ = 1.3 points above CG_post (*p* = .104). Concerning the age variable in the Static-99R, it was found that the three study groups did not differ in this respect (see Table 1).

### Diagnoses According to ICD-10 and Criminal Responsibility

**Figure 3.**
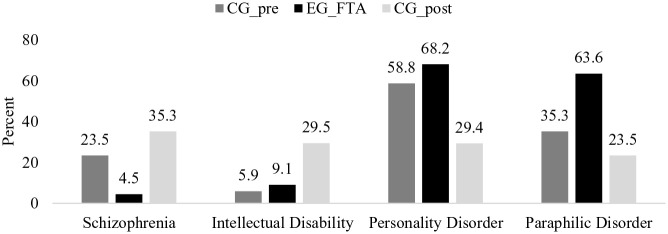
Diagnoses of the Comparison Group released prior to the foundation of the FTA (CG_pre; *n* = 17), Treatment Group FTA (TG_FTA; *n* = 22) and the Comparison Group released after the foundation of the FTA but without FTA-treatment (CG_post; *n* = 17). *Note.* As some persons had been given more than one diagnosis, the totals do not add up to 100%.

The diagnoses given during their treatment under the last HTO did not show any significant differences between the persons released prior to the foundation of the FTA (CG_pre) and the treatment group TG_FTA (see Figure 3; Schizophrenia: CG_pre *n* = 4 of 17, TG_FTA *n* = 1 of 22, *Fisher’s Exact Test* = 3.185, *ϕ* = .28, *p* = .147; Intellectual Disability: CG_pre *n* = 1 of 17, TG_FTA *n* = 2 of 22, *Fisher’s Exact Test* = 0.139, *ϕ* = −.06, *p* = 1.000; Personality Disorder: CG_pre *n* = 10 of 17, TG_FTA *n* = 15 of 22, *χ*^2^_(1,39) = _0.365, *ϕ* = −.10, *p* = .783; Paraphilic Disorder: CG_pre *n* = 6 of 17, TG_FTA *n* = 14 of 22, *χ*^2^_(1,39) = _3.083, *ϕ* = −.28, *p* = .111). A closer look at the numbers reveals that quite high effect sizes were achieved in terms of Schizophrenia (*ϕ* = .28) and paraphilic disorders (*ϕ* = −.28).

Between the study groups released since the foundation of the FTA, there were marked differences regarding the diagnoses (see Figure 3). In the treatment group TG_FTA were significantly fewer persons with a diagnosed schizophrenia (TG_FTA *n* = 1 of 22, CG_post *n* = 6 of 17, *Fisher’s Exact Test* = 6.498, *ϕ* = .40, *p* = .030), but more persons with diagnosed personality disorders (TG_FTA *n* = 15 of 22, CG_post *n* = 5 of 17, *χ*^2^_(1,39) = _5.770, *ϕ = −*.59, *p* = .025) and paraphilic disorders (TG_FTA *n* = 14 of 22, CG_post *n* = 4 of 17, *χ²*_(1,39)_ = 6.207, *ϕ* = −.40, *p* = .023) than in the CG_post. With an effect size of *ϕ* = .26, there was no significant difference between the two study groups with respect to Intellectual Disability (TG_FTA *n* = 2 of 22, CG_post *n* = 5 of 17, *Fisher’s Exact Test* = 2.707, *ϕ* = .26, *p* = .205). Due to the small number, no calculations were performed for the different paraphilic disorders. When looking at the numbers, however, it is noticeable that persons who had been diagnosed with sexual sadism disorder were all included in the treatment group TG_FTA (CG_pre: *n* = 0; TG_FTA: *n* = 2; CG_post: *n* = 0). In contrast, almost the same distribution was found between the three study groups for persons diagnosed with a pedophilic disorder (CG_pre: *n* = 5; TG_FTA: *n* = 5; CG_post: *n* = 4).

**Figure 4.**
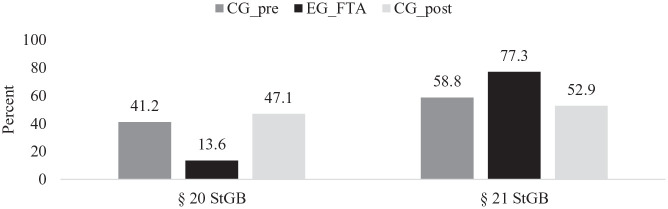
Limited criminal responsibility within the meaning of criminal law of the comparison group released prior to the foundation of the FTA (CG_pre; *n* = 17), Treatment Group FTA (TG_FTA; *n* = 22) and the Comparison Group released after the foundation of the FTA but without FTA-Treatment (CG_post; *n* = 17). *Note.* § 20 StGB (Strafgesetzbuch; German Criminal Code): Incapable of Contracting Guilt within the meaning of German Criminal Law; § 21 StGB: Diminished Responsibility of Contracting Guilt within the meaning of German Criminal Law.

Overall, the number of persons released via the FTA who were found incapable of contracting guilt within the meaning of German Criminal Law according to § 20 StGB (Strafgesetzbuch; German Criminal Code) was lower (see Figure 4). While the difference to the CG_pre was not significant (CG_pre: *n* = 7 of 17, TG_FTA: *n* = 3 of 22, *Fisher’s Exact Test* = 3.843, *ϕ* = −.31, *p* = .071), there was a significant difference to the CG_post (CG_post: *n* = 8 of 17, TG_FTA: *n* = 3 of 22, *Fisher’s Exact Test* = 5.368, *ϕ* = −.37, *p* = .033). Although the TG_FTA contained more persons classified by court as diminished responsibility of contracting guilt within the meaning of German Criminal Law according to § 21 StGB, this difference was not significant compared to either CG_pre (CG_pre: *n* = 10 of 17, TG_FTA: *n* = 17 of 22, *χ²*_(1,39) = _1.532, *ϕ* = .20, *p* = .299), or the CG_post (CG_post: *n* = 9 of 17, TG_FTA: *n* = 3 of 22, *χ*^2^_(1,39) = _2.555, *ϕ* = .26, *p* = .172

## Discussion

Although the current state of research may be optimistic, the effectiveness of forensic outpatient follow-up treatment regarding the criterion of recidivism cannot yet be substantiated due to methodological difficulties (Sauter et al., 2017; Schmucker & Lösel, 2017). Since RCTs will probably not be available in the near future, other ways must be explored to examine what works how, when and for whom. As it will not always be possible to focus on the criterion variable of recidivism, it is even more important to focus on the conditions as well as the implementation and organization of the forensic follow-up treatment as well as other outcomes. Research on this topic is of tremendous relevance due to its public safety dimension (Basile et al., 2007; Collin-Vézina et al., 2013).

Thus, the aim of the present study was to investigate whether the foundation of the Forensic Therapeutic Outpatient Clinic (FTA) in Berlin, Germany, was associated with an improvement in the release prospects for persons who had committed sexual offenses. It was hypothesized that with the availability of professionalized forensic follow-up treatment, the risk tolerance of the participating bodies increased and, accordingly, the group released post-foundation and via the FTA showed an overall higher initial risk. When considering the most objective criterion, the well-validated Static-99R risk assessment tool (Phenix et al., 2016), this question can be answered in the affirmative. The group released prior to the foundation of the FTA (CG_pre) showed the lowest scores, while those released with FTA follow-up treatment (TG_FTA) showed the highest scores and thus the highest initial risk. Since no significant differences in age were observed between the study groups at the time of release, this effect cannot be attributed to this either (Hanson, 2002). In fact, the CG_pre was the youngest study group on average.

Regarding other criteria examined in this study, however, this group difference was not so clearly evident. Thus, there were hardly any differences between the CG_pre and TG_FTA, but since the foundation of the FTA, those with the significantly more serious previous criminal history and the more serious index offenses do not seem to be released without professionalized forensic follow-up treatment in Berlin anymore. For instance, there were no significant differences in the type of index offense between the three research groups, but after the foundation of the FTA, persons who had committed a sexually motivated homicide were no longer released without forensic follow-up treatment via the FTA while they had been released as well prior to the foundation of the FTA. While the duration of the custodial sentence imposed by the court for the index offense did not differ significantly between CG_pre and TG_FTA, the custodial sentence imposed was significantly lower for those released after the FTA was founded but without its support (CG_post). Since the prison sentence can be seen as a measure of the seriousness of the offenses committed, professionalized forensic follow-up treatment by the FTA seems to have been used for patients potentially at a higher risk of reoffending.

A very similar result was obtained when examining the criminal history of the three study groups in detail: Overall, the persons released via the FTA (TG_FTA) were the youngest at the time of their first offense, at the time of the index offense they had the most previous convictions and the longest previous prison experience. The highest age at their first offense, the lowest number of previous convictions and the shortest previous prison experience had those, who were released after the foundation of the FTA but without FTA-Treatment (CG_post). The values for those released before the FTA was founded (CG_pre) lay between these two groups, with significant differences only between the two study groups released after the FTA was founded. It is therefore not surprising that the persons of the TG_FTA had the longest treatment duration under a HTO with over 16 years. While the persons of the CG_pre with an average of 8.5 years were treated significantly shorter under an HTO, with almost 13 years there was no significant difference to the CG_post. According to the lowest probability of recidivism measured by Static-99R in the CG_pre, it is quite possible that according to the RNR-principles (Andrews & Bonta, 2010) in this group a release from the potentially unlimited measure of the HTO became possible most quickly. In view of the long retrospective study duration, however, this could also be a cohort effect: While in 1998 in Germany there was still a general reduction in the treatment duration under a HTO (Leygraf, 1998), in 2006 it was noted that the average treatment duration had risen to about 6 years, while the number of releases persons had fallen considerably (Leygraf, 2006).

No group differences were found between the CG_pre and the TG_FTA regarding the diagnostic assessment according to ICD-10. Although more persons released prior to the foundation of the FTA were diagnosed with schizophrenia and fewer were diagnosed with a paraphilic disorder, this difference was not significant. It is worth noting, however, that high effect sizes were recorded in this respect. The situation is different with the two study groups released after the FTA was founded. Professional forensic follow-up treatment via the FTA was implemented significantly less frequently in patients with schizophrenia who had committed a sexual offense, but significantly more frequently in patients with personality disorders and paraphilic disorders who had committed a sexual offense. Presumably, those in whom the cause of the sexual offense could be seen in the schizophrenic disorder were more likely to be released through psychiatric community follow-up treatment structures. Despite the very small number of cases, it can be noted that the two persons who had been diagnosed with sexual sadism were released via the FTA, while persons with a diagnosed pedophilic disorder were found in all three study groups. In accordance with the diagnostic differences described, there was no significant difference with regard to a diminished responsibility of contracting guilt within the meaning of German Criminal Law (§ 21 StGB; German Criminal Code). However, significantly fewer people incapable of contradicting guilt within the meaning of German Criminal Law (§ 20 StGB, German Criminal Code; that is, persons suffering from schizophrenia) were released via the FTA.

Our results pose different implications for both policy and practice. Forensic aftercare treatment seems to be effective in shaping the releasability of persons who committed sexual offenses. That result is a promising finding in terms of lowering overpopulation in forensic institutions (Leygraf, 2006) and in terms of lowering costs that are produced by a prolonged stay within forensic institutions (Cohen & Piquero, 2009). Costs that are saved within these institutions could be invested into the forensic aftercare facilities themselves (Butz et al., 2013) to provide a high-quality treatment for forensic outpatients. The available evaluations indicate the effectiveness of forensic aftercare in reducing recidivism (Sauter et al., 2017; Schmucker & Lösel, 2017), although it remains to be seen whether this is achieved through treatment or through its monitoring function.

### Limitations

The following limitations should be considered: The sample size is statistically speaking small. However, the data presented are a complete survey of all persons who had committed a sexual offense and could be released from forensic hospital by a court in Berlin since 1997. As opposed to randomized controlled trial studies the retrospective design of this study is more susceptible to possible confounding effects (e.g., the cohort effect described above). Therefore, and because of the non-random assignment to the different groups invested, it is not possible to fully disentangle whether individuals with an HTO were released earlier because of the available treatment from the professionalized forensic outpatient clinic or because of different reasons. Also, with the Static-99R only one validated risk assessment tool for sexual offenders was used. However, it is the most commonly used (Helmus & Hanson, 2007) and most suitable tool for the group of persons who offended sexually.

## Conclusion

The aim of establishing forensic outpatient clinics was to improve release options to enable those who are difficult to release to be released and thus counteract the overcrowding of forensic hospitals (Freese, 2003; Leygraf, 2006). In this study, it was indeed found that persons who had committed a sexual offense and showed a high initial risk were released via the FTA. Both, Static-99R scores and treatment duration were highest in this group. Due to the methodological difficulties, the extent to which these persons were released only through professionalized forensic follow-up treatment must remain an open question. Those who were released before the FTA was founded were not at the lowest risk in terms of the criminogenic markers recorded, but rather at medium risk. This also suggests that since the establishment of the FTA, this additional option has been used more frequently and people who were previously released without this multi-professional treatment are now receiving support from the FTA. A circumstance that can also be viewed critically in view of the not uncommon involuntary nature of this measure.
